# Social Media Use and Health-Related Quality of Life Among Adolescents: Cross-sectional Study

**DOI:** 10.2196/39710

**Published:** 2022-10-04

**Authors:** Yueyue You, Junwen Yang-Huang, Hein Raat, Amy Van Grieken

**Affiliations:** 1 The Generation R Study Group Erasmus Medical Center Rotterdam Netherlands; 2 Department of Public Health Erasmus Medical Center Rotterdam Netherlands

**Keywords:** adolescents, social media platforms, social media, health-related quality of life, EuroQol 5-dimension questionnaire, youth version

## Abstract

**Background:**

Using social media is a time-consuming activity of children and adolescents. Health authorities have warned that excessive use of social media can negatively affect adolescent social, physical, and psychological health. However, scientific findings regarding associations between time spent on social media and adolescent health-related quality of life (HRQoL) are not consistent. Adolescents typically use multiple social media platforms. Whether the use of multiple social media platforms impacts adolescent health is unclear.

**Objective:**

The aim of this study was to examine the relationship between social media use, including the number of social media platforms used and time spent on social media, and adolescent HRQoL.

**Methods:**

We analyzed the data of 3397 children (mean age 13.5, SD 0.4 years) from the Generation R Study, a population-based cohort study in the Netherlands. Children reported the number of social media platforms used and time spent on social media during weekdays and weekends separately. Children’s HRQoL was self-reported with the EuroQol 5-dimension questionnaire–youth version. Data on social media use and HRQoL were collected from 2015 to 2019. Multiple logistic and linear regressions were applied.

**Results:**

In this study, 72.6% (2466/3397) of the children used 3 or more social media platforms, and 37.7% (1234/3276) and 58.3% (1911/3277) of the children used social media at least 2 hours per day during weekdays and weekends, respectively. Children using more social media platforms (7 or more platforms) had a higher odds of reporting having some or a lot of problems on “having pain or discomfort” (OR 1.55, 95% CI 1.20 to 1.99) and “feeling worried, sad or unhappy” (OR 1.99, 95% CI 1.52 to 2.60) dimensions and reported lower self-rated health (β –3.81, 95% CI –5.54 to –2.09) compared with children who used 0 to 2 social media platforms. Both on weekdays and weekends, children spent more time on social media were more likely to report having some or a lot of problems on “doing usual activities,” “having pain or discomfort,” “feeling worried, sad or unhappy,” and report lower self-rated health (all *P*<.001).

**Conclusions:**

Our findings indicate that using more social media platforms and spending more time on social media were significantly related to lower HRQoL. We recommend future research to study the pathway between social media use and HRQoL among adolescents.

## Introduction

The number of adolescents who use social media daily has doubled in western countries, from 34% in 2010 to 70% in 2020 [1]. Social media use can be defined as using various media platforms, such as Facebook, Twitter, YouTube, and so on, to quickly create and share content with the public [2]. Data suggest that in the European countries, 92% of children aged 14 to 16 years use social media and 39% visit social media platforms at least once a day [3]. Using social media is among the most common activities of today’s children [4-6]. Meanwhile, extensive social media use may impact children’s physical and psychosocial well-being [7,8].

Findings on whether social media use improves or reduces children’s well-being are inconsistent [6,9]. Engagement via social media platforms provides opportunities for keeping in touch with families and friends and other social interactions that may increase children’s emotional well-being [10]. Social media can also be a valuable resource for peer support, allowing children to get advice from others or share their own experiences [11,12]. By expanding the quantity and quality of communications, social media use enhances children’s social well-being [13].

However, studies have also shown that excessive social media use might decrease children’s well-being [14,15]. For example, one study among children suggested that excessive social media use may expose them to idealized depictions of others [16]. This may trigger negative social comparisons that cause users to believe others are happier and have better lives, increasing anxiety and decreasing their mental well-being [17,18]. Moreover, Nie et al [19] suggested that more time spent on social media may lead to less time spent on health-promoting behaviors such as physical activity, reducing the children’s physical well-being. Studies have started to evaluate the number of social media platforms (ie, Facebook, Instagram) that a person uses [20,21], since adolescents do not typically use just one social media platform; more than 70% use multiple social media platforms [22]. Previous studies have shown that people who use multiple platforms are more likely to be exposed to news or posts about others’ successes, which may increase users’ dissatisfaction with their own lives and thus decrease well-being [23-25]. Overall, the inconsistent findings on a broad range of child health outcomes suggest more research is needed. Specifically, health-related quality of life (HRQoL) might be a relevant outcome to study in order to evaluate the impact of the use of social media platforms on children’s health and well-being [26]. HRQoL is a multidimensional concept, including disease state and physical, psychological, and social well-being [27].

The aim of this study was to examine the relationship between the number of social media platforms used, time spent on social media, and HRQoL among children aged 13 years. We hypothesized that children who use multiple social media platforms and spend more time on social media would be likely to have lower HRQoL.

## Methods

### Design and Study Population

**Figure 1:**
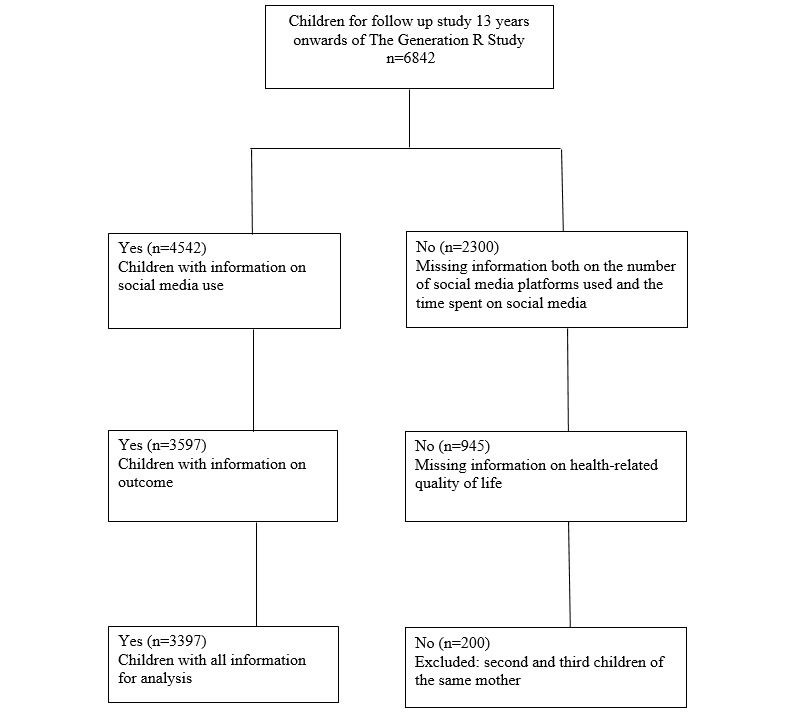
Flowchart of participants.

A cross-sectional study was designed using date from a cohort study—the Generation R Study. The Generation R Study is an ongoing prospective cohort study from fetal life onward in Rotterdam, The Netherlands. Detailed information on the Generation R Study has been published elsewhere [28]. Briefly, all pregnant women with an expected delivery date from April 2002 through January 2006 living in Rotterdam were invited to participate. A total of 6842 children participated in the assessment at age 13 years (2015-2019). Children with missing data on the number of social media platforms used or the time spent on social media were excluded (n=2300), as were children with missing data on HRQoL (n=945). To avoid data clustering, second (n=192) and third children (n=8) of the same mother were excluded, leaving a study population of 3397 children (Figure 1). Written informed consent was obtained from all participants.

### Ethics Approval

The Medical Ethics Committee of the Erasmus University Medical Center approved the study (MEC 217.595/2002/202).

### Measures

#### Exposure Variables

Children were asked to report which social media platforms they used. Nine widely used social media platforms (ie, Facebook, Instagram, Musical.ly, Pinterest, Skype, Snapchat, Twitter, WhatsApp, YouTube), “Other, namely:_,” and never used (ie, I do not use these platforms) were listed as the response options. To operationalize this variable, the number of different platforms used was counted. The variable was then divided into 0 to 2 platforms, 3 to 4 platforms, 5 to 6 platforms, and 7 or more platforms.

The time spent on social media was examined separately on weekdays and weekends since children spend more time on their phones or other devices at the weekends than on weekdays, as they are at school during the weekdays [29]. The time spent on social media was measured by self-reported questionnaire. Children gave answers to the following questions: “On average, how many hours per day do you usually spend on social media during weekdays?” and “On average, how many hours per day do you usually spend on social media during weekend days?” Response options for these questions were <30 minutes, 30 minutes to 2 hours, 2 to 4 hours, 4 to 6 hours, and >6 hours.

#### Outcome Variable

Children’s HRQoL was measured using EuroQol 5-dimension questionnaire–youth version (EQ-5D-Y) developed by the European Quality of Life Group [30]. The EQ-5D-Y questionnaire consists of 2 parts to measure the generic health of children: the EQ-5D descriptive system and the visual analog scale (VAS). Children were asked to rate their health in the present day on 5 dimensions: “mobility” (refers to physical ability to walk or move about, both inside and outside), “looking after myself” (refers to an age-appropriate degree of independence in daily personal care, specifically covering washing and dressing), “doing usual activities” (refers to the ability to participate in child-specific activities, such as going to school, hobbies, sports, playing, and doing things with family or friends), “having pain or discomfort” (refers to physical/bodily soreness or uncomfortable physical sensation of a lower grade of intensity than pain such as aches, nausea, dizziness), and “feeling worried, sad or unhappy.” Children could choose from 3 levels (no problems, some problems, or a lot of problems) to answer each dimension. Because few children chose “a lot of problems” in each dimension, the 3 levels under each dimension were collapsed into 2 levels in this study (no problems and some or a lot of problems) [31]. The VAS records children’s self-rated health (“How good is your health TODAY?”) on a vertically numbered VAS score from 0 to 100. Zero was labeled as “the worst imaginable health state” while 100 was labeled as “the best imaginable health state.” The feasibility, validity, and reliability of the EQ-5D-Y have been documented [32].

#### Covariates

Based on the literature, several variables were considered as the potential confounders in this study: child’s age, sex, ethnic background, family composition, net household income, and maternal educational level. Child ethnic background (Western, non-Western) was based on parental countries of birth obtained from questionnaire when the child was aged 6 years [33]. Family composition (1-parent family, 2-parent family) and net household income per month (<€2000 [US $1933] per month, €2000-€3600 [US $1933-$3479] per month, >€3600 [US $3479] per month) were obtained by parent-report questionnaire when the child was aged 13 years. Maternal educational level was obtained when the child was aged 6 years by parent-report and was defined by the highest education attained. It was divided into 3 categories: low, middle, and high.

### Statistical Analyses

Descriptive analyses were applied to characterize the study population. Distribution of the reported problems in the 5 dimensions of HRQoL by the number of social media platforms used and the time spent on social media were assessed by chi-square tests and shown in Multimedia Appendix 1. The VAS score by the number of social media platforms used and the time spent on social media were evaluated by 1-way analysis of variance and shown in Multimedia Appendix 1. The relationship between the number of social media platforms used and HRQoL was investigated using logistics regression for the 5 dimensions and linear regression for VAS score. All models were adjusted for the child’s age, sex, ethnic background, family composition, maternal educational level, and net household income and additionally adjusted for time spent on social media. The relationship between the time spent on social media and HRQoL was investigated using logistics regression for 5 dimensions and linear regression for VAS score and shown in Multimedia Appendix 2. All models were adjusted for the covariates. All models were run separately with weekday and weekend social media use.

Missing data on covariates were imputed using multiple imputation methods, and 10 imputed data sets were generated. Pooled effect estimates (odds ratios [ORs] and β coefficients) and confidence intervals from these 10 imputed data sets were reported, and *P*<.05 was used to indicate statistical significance. Statistical analyses were performed using SPSS for Windows (version 24.0, IBM Corp).

### Nonresponse Analyses

Children with missing data on HRQoL (n=945) were compared with children without missing data (n=3397) using chi-square tests. Data were more often missing for children from mothers with a low educational level, a low household income, or a 1-parent family (all *P*<.05). No statistical difference was found in the number of social media platforms used and time spent on social media between children with or without data on HRQoL.

### Sensitivity Analyses

To examine the robustness of our results, the relationship between the number of social media platforms used and HRQoL was examined using the continuous variable of the number of social media platforms used and shown in Multimedia Appendix 3.

Additionally, one variable-parental supervision was added as a confounder into the regression models and shown in Multimedia Appendices 4 and 5. This variable was obtained by a parent-reported questionnaire when the child was aged 13 years. Parents answered the question “I sit at the computer together with my child if they are on social media.” Response options were never, rarely, sometimes, often, and always.

## Results

### Sample Characteristics

In total, 3397 children were included in this study. The mean age of the children was 13.5 (SD 0.4) years; 46.9% (1594/3397) were boys. More than two-thirds (2466/3397, 72.6%) of the children used 3 or more social media platforms. Of these, 37.7% (1234/3276) of the children used social media more than 2 hours on a weekday and 58.3% (1911/3277) did on a weekend day. From all 5 dimensions in HRQoL, children reported the most problems in “having pain or discomfort” (876/3385, 25.9%), followed by “feeling worried, sad, or unhappy” (557/3388, 16.4%). The average VAS score was 83.5 (SD 14.8; Table 1).

**Table 1:** General characteristics of the study population (n=3397).

Characteristic				Total			Missing
Child’s age (years), mean (SD)				13.5 (0.4)			112 (3.3)
Child’s sex, boy, n (%)				1594 (46.9)			0
**Child’s ethnic background, n (%)**							
	Western			2557 (76.0)		33 (1.0)	
	Non-western			807 (24.0)		—^a^	
**Maternal education level, n (%)**							
	Low			257 (8.2)		268 (7.9)	
	Middle			860 (27.5)		—	
	High			2012 (64.3)		—	
**Household income (€) per month, n (%)**							
	<2000			411 (13.7)		398 (11.7)	
	2000-3600			791 (26.4)		—	
	>3600			1797 (59.9)		—	
**Family composition, n (%)**							
	1-parent family			542 (16.7)		149 (4.4)	
	2-parent family			2706 (83.3)		—	
**Number of social media platforms used, n (%)**							
	0-2			931 (27.4)		0	
	3-4			1154 (34.0)		—	
	5-6			763 (22.5)		—	
	≥7			549 (16.2)		—	
**Time spent on social media**							
	**Weekdays (hours), n (%)**						
		<0.5	409 (12.5)		121 (3.6)		
		0.5-2	1633 (49.8)		—		
		2-4	989 (30.2)		—		
		4-6	164 (5.0)		—		
		>6	81 (2.5)		—		
	**Weekend days (hours), n (%)**						
		<0.5	251 (7.7)		120 (3.5)		
		0.5-2	1115 (34.0)		—		
		2-4	1408 (43.0)		—		
		4-6	321 (9.8)		—		
		>6	182 (5.6)		—		
**Health-related quality of life**							
	**Mobility, n (%)**						
		No problems	3199 (94.4)		7 (0.2)		
		Some or a lot of problems	191 (5.6)		—		
	**Looking after myself, n (%)**						
		No problems	3340 (98.5)		6 (0.2)		
		Some or a lot of problems	51 (1.5)		—		
	**Doing usual activities, n (%)**						
		No problems	3155 (93.1)		8 (0.2)		
		Some or a lot of problems	234 (6.9)		—		
	**Having pain or discomfort, n (%)**						
		No problems	2509 (74.1)		12 (0.4)		
		Some or a lot of problems	876 (25.9)		—		
	**Feeling worried, sad, or unhappy, n (%)**						
		No problems	2831 (83.6)		9 (0.3)		
		Some or a lot of problems	557 (16.4)		—		
	EQ^b^ VAS^c^, mean (SD)			83.5 (14.8)		538 (15.8)	

^a^Not applicable.

^b^EQ: EuroQoL.

^c^VAS: visual analog scale.

Multimedia Appendix 1 shows the distribution of reported problems in the 5 dimensions and VAS score by the number of social media platforms used and the time spent on social media. Significant differences were found in “mobility,” “doing usual activities,” “having pain or discomfort,” and “feeling worried, sad or unhappy” dimensions between children who spent different time on social media (all *P*<.05). The VAS score decreased with more social media platforms used and with more time spent on social media.

### Number of Social Media Platforms Used and HRQoL

Compared with children who used 0 to 2 platforms, those who used 5 to 6 platforms or 7 or more platforms were more likely to report having some or a lot of problems on “having pain or discomfort” dimension (5-6 platforms: OR 1.41, 95% CI 1.12 to 1.78; 7 or more platforms: OR 1.55, 95% CI 1.20 to 1.99). Compared with children who used 0 to 2 platforms, those who used more platforms were more likely to report having some or a lot of problems on “feeling worried, sad or unhappy” dimension (3-4 platforms: OR 1.42, 95% CI 1.09 to 1.85; 5-6 platforms: OR 1.61, 95% CI 1.25 to 2.08; 7 or more platforms: OR 1.99, 95% CI 1.52 to 2.60). Furthermore to using 7 or more social media platforms was related to lower VAS score (β –3.81, 95% CI –5.54 to –2.09; Table 2).

**Table 2:** The relationship between the number of social media platforms used and health-related quality of life among children aged 13 years.

HRQoL^a^	0-2 platforms	3-4 platforms	5-6 platforms	7≥ platforms
Mobility, OR^b^ (95% CI)	1 (ref)	1.04 (0.70 to 1.54)	1.24 (0.81 to 1.89)	0.94 (0.58 to 1.53)
Looking after myself, OR (95% CI)	1 (ref)	0.55 (0.27 to 1.13)	0.61 (0.27 to 1.38)	0.75 (0.32 to 1.76)
Ding usual activities, OR (95% CI)	1 (ref)	0.72 (0.51 to 1.02)	0.70 (0.47 to 1.04)	1.03 (0.69 to 1.53)
Having pain or discomfort, OR (95% CI)	1 (ref)	1.23 (0.99 to 1.52)	1.41 (1.12 to 1.78)	1.55 (1.20 to 1.99)
Feeling worried, sad or unhappy, OR (95% CI)	1 (ref)	1.42 (1.09 to 1.85)	1.61 (1.25 to 2.08)	1.99 (1.52 to 2.60)
EQ^c^ VAS^d^, 𝛽 (95% CI)	1 (ref)	–0.35 (–1.74 to 1.03)	–0.91 (–2.45 to 0.63)	–3.81 (–5.54 to –2.09)

^a^HRQoL: health-related quality of life.

^b^OR: odds ratio.

^c^EQ: EuroQoL.

^d^VAS: visual analog scale.

### Time Spent on Social Media and HRQoL

After adjusting for all variates, higher social media use both on weekdays and weekends were related to more problems reported in each dimension and lower VAS score (Multimedia Appendix 2). On a weekday, compared to children who used social media less than 30 minutes per day, children who used social media between 2 to 4 hours, 4 to 6 hours, or more than 6 hours were more likely to report having some or a lot of problems on 3 dimensions (“doing usual activities,” “having pain or discomfort” and “feeling worried, sad, or unhappy” dimensions; all *P*<.05). Of those 3 dimensions, children who used social media more than 6 hours reported the highest odds ratio of reporting problems on “doing usual activities” dimension compared to children who used social media less than 30 minutes per day (OR 4.00, 95% CI 1.84 to 8.71). On the “mobility” dimension, only children who used social media more than 6 hours reported significantly more problems compared to those who used social media less than 30 minutes (OR 2.21, 95% CI 1.05 to 4.65).

With regard to the VAS score, higher social media use was related to lower VAS score. Children who used social media more than 6 hours per day had the lowest VAS scores compared with children who used less than 30 minutes per day (β –7.70, 95% CI –11.82 to –3.59). The results of the relationship between the time spent on social media and HRQoL during weekends were comparable to the results of weekdays, although effect estimates (ORs and β) were smaller.

### Findings From Sensitivity Analyses

The results showed that using more social media platforms was related to reported problems on the “having pain or discomfort” (OR 1.10, 95% CI 1.04 to 1.17) and “feeling worried, sad or unhappy” (OR 1.11, 95% CI 1.03 to 1.18) dimensions and lower VAS score (β –0.42, 95% CI –0.81 to –0.03) when regarding the number of social media platforms used as the continuous variable (Multimedia Appendix 3). Additionally adjusting for the confounder parental supervision, all the results were comparable to previous analyses, although effect estimates (ORs and β) were smaller (Multimedia Appendices 4 and 5).

## Discussion

### Principal Findings

This study investigated the relationship between the number of social media platforms used, time spent on social media, and HRQoL in children aged 13 years. The findings show that 72.6% of the children aged 13 years used 3 or more social media platforms. Around 40% of the children used social media at least 2 hours per day during weekdays (37.7%) and over half of the children on weekends (58.3%). Our findings also show that using more social media platforms and spending more time on social media were related to lower HRQoL.

### Number of Social Media Platforms Used and HRQoL

A higher number of social media platforms used was related to lower HRQoL, which is in line with our hypothesis. Children who used more social media platforms had a higher odds of reporting having some or a lot of problems on the 5 HRQoL dimensions and reported lower self-rated health than their counterparts. An increased number of social media platforms used may elevate the stress of meeting the expectations to check for updates and respond on time on several social media platforms [34]. Moreover, being bombarded with information and communication from numerous social media platforms may result in media multitasking problems [35]. Media multitasking is a specific type of media use behavior in which users simultaneously perform at least 2 media activities or frequently change from one media activity to another. The act may decrease children’s psychological well-being, self-esteem, and overall HRQoL [36].

However, it is important to note that decreasing the number of social media platforms used for children may be challenging in today’s world. Children may be reluctant to give up any platform because they use different platforms for different reasons [37]. For example, a child can have a Facebook account to keep in touch with friends, Pinterest for cooking, Twitter for news, and Instagram for blogging. Therefore, educational intervention may help adolescents, especially those beginning to use social media, to better understand which platforms are truly necessary to their lives and valuable for their goals and which ones may not be [38].

### Time Spent on Social Media and HRQoL

Our findings suggest that more social media use (on both weekdays and weekends) was related to a lower HRQoL, which is in line with the hypothesis. Children who spent more time on social media were more likely to report having some or a lot of problems on the 5 HRQoL dimensions, and report lower self-rated health than those who spent less time on social media. These findings are in line with previous studies demonstrating that social media use among children aged 13 years and older was inversely associated with HRQoL outcomes such as physical health status and psychological well-being [39,40].

Previous studies have shown that children who spend more time on social media might have a greater chance of being the victim of cyberbullying or direct attacks from others on their sense of well-being [41]. Sampasa et al [42] reported that social media use was associated with cyberbullying victimization and that the risk of being cyberbullied increased in a dose-response manner in children. Children who suffer from cyberbullying are more likely to have lower HRQoL [43]. On the other hand, studies also reported that children who had limited activities and/or high levels of feeling worried, sad or unhappy may perceive online social communication via social media as a possibility to gain social support and relieve themselves of negative feelings through the ease of online self-disclosure [44]. Future studies are recommended to study intermediate variables to better understand the pathway between adolescents’ social media use and HRQoL.

### Strengths and Limitations

In this study, social media use was assessed with the number of social media platforms used and time spent on social media. Further, this study was conducted among children aged 13 years, a sensitive developmental period during which little is known about social media use and its impact on HRQoL. However, the following methodological considerations need to be taken into account. First, for our cross-sectional analyses data from children aged 13 years were used. During this measurement, data on time spent on social media and the type of social media used were collected. The next measurement was performed on child aged 18 years. We recommended follow-up studies to explore the longitudinal associations between social media use and HRQoL. Second, social media use was captured via self-reported questionnaire, which may lead to overestimating or underestimating the time spent on social media. More accurate measuring method such as passive sensing (eg, apps installed on the devices that track real-time use [45]), could be adopted in future studies.

### Conclusions

This study captured the number of social media platforms used and time spent on social media among children aged 13 years. Children using more social media platforms and spending more time on social media were more likely to report a lower HRQoL, including reporting having some or a lot of problems on the 5 HRQoL dimensions and lower self-rated health. Better understanding of how social media platforms contribute to children’s health and well-being may contribute to more support in adolescent social media use.

## Appendix

Multimedia Appendix 1 Distribution of the reported problems in the 5 dimensions of HRQoL and the VAS score by the number of social media platforms used and the time spent on social media among children aged 13 years.

Multimedia Appendix 2 The relationship between the time spent on social media and health-related quality of life among children aged 13 years.

Multimedia Appendix 3 Sensitivity analyses: the relationship between the number of social media platforms used and health-related quality of life.

Multimedia Appendix 4 Sensitivity analyses: the relationship between the number of social media platforms used and health-related quality of life.

Multimedia Appendix 5 Sensitivity analyses: the relationship between the time spent on social media and health-related quality of life.

## Abbreviations

EQ-5D-Y: EuroQol 5-dimension questionnaire–youth version
HRQoL: health-related quality of life
OR: odds ratios
VAS: visual analog scale
