# Supplementary material for: Social Media Use and Health-Related Quality of Life Among Adolescents: Cross-sectional Study
Source: JMIR Ment Health. 2022 Oct 4;9(10):e39710. doi: 10.2196/39710 (PMC9579926; doi:10.2196/39710)
Supplement: Multimedia Appendix 1 [file mental_v9i10e39710_app1.docx]

Multimedia Appendix 1. Distribution of HRQoL by the number of social media platforms used and the time spent on social media among children aged 13 years.

|  | Mobility^a^  n (%) | Looking after myself^a^  n (%) | Doing usual activities^a^  n (%) | Having pain or discomfort^a^  n (%) | Feeling worried,  sad or unhappy^a^  n (%) | EQ VAS^b^  mean (SD) |
| --- | --- | --- | --- | --- | --- | --- |
| **The number of social media platforms used** |  |  |  |  |  |  |
| 0 to 2 platforms | 48 (25.1) | 21 (41.2) | 74 (31.6) | 186 (21.2) | 133 (23.9) | 84.40 (14.35) |
| 3 to 4 platforms | 63 (33.0) | 13 (25.5) | 68 (29.1) | 290 (33.1) | 140 (25.1) | 84.05 (14.26) |
| 5 to 6 platforms | 51 (26.7) | 9 (17.6) | 45 (19.2) | 225 (25.7) | 152 (27.3) | 83.49 (14.59) |
| 7 or platforms | 29 (15.2) | 8 (15.7) | 47 (20.1) | 175 (20.0) | 132 (23.7) | 80.59 (16.72) |
| *P*-vaule | .526 | .157 | .075 | **<.001** | **<.001** | **<.001** |
| **The time spent on social media, weekday** |  |  |  |  |  |  |
| < 30 min | 27 (14.8) | 1 (2.0) | 17 (7.7) | 84 (9.9) | 46 (8.7) | 85.55 (14.15) |
| 30 min to 2 hours | 74 (40.7) | 24 (48.0) | 102 (45.9) | 393 (46.5) | 248 (46.8) | 84.15 (14.72) |
| 2 to 4 hours | 58 (31.9) | 21 (42.0) | 74 (33.3) | 280 (33.1) | 181 (34.2) | 82.60 (14.30) |
| 4 to 6 hours | 11 (6.0) | 4 (8.0) | 16 (7.2) | 56 (6.6) | 36 (6.8) | 79.84 (17.92) |
| > 6 hours | 12 (6.6) | 0 (0) | 13 (5.9) | 33 (3.9) | 19 (3.6) | 76.79 (16.42) |
| *P*-vaule | **.001** | .060 | **<.001** | **<.001** | **<.001** | **<.001** |
| **The time spent on social media, weekend day** |  |  |  |  |  |  |
| < 30 min | 13 (7.1) | 1 (2.0) | 13 (5.8) | 49 (5.8) | 39 (7.3) | 85.71 (14.70) |
| 30 min to 2 hours | 55 (30.1) | 10 (20.4) | 64 (28.6) | 268 (31.6) | 158 (29.7) | 84.19 (15.11) |
| 2 to 4 hours | 84 (45.9) | 27 (55.1) | 98 (43.8) | 364 (42.9) | 234 (44.0) | 83.43 (14.12) |
| 4 to 6 hours | 9 (4.9) | 7 (14.3) | 28 (12.5) | 96 (11.3) | 54 (10.2) | 81.08 (15.43) |
| > 6 hours | 22 (12.0) | 4 (8.2) | 21 (9.4) | 71 (8.4) | 47 (8.8) | 79.21 (16.36) |
| *P*-vaule | **<.001** | .087 | **.023** | **<.001** | **.003** | **<.001** |

The table is based on a nonimputed data set.

^a^ *P*-value are calculated by Chi-square tests.

^b^ *P*-value are calculated by ANOVA.
