# Supplementary material for: Social Media Use and Health-Related Quality of Life Among Adolescents: Cross-sectional Study
Source: JMIR Ment Health. 2022 Oct 4;9(10):e39710. doi: 10.2196/39710 (PMC9579926; doi:10.2196/39710)
Supplement: Multimedia Appendix 2 [file mental_v9i10e39710_app2.docx]

Multimedia Appendix 2. The relationship between the time spent on social media and health-related quality of life among children aged 13 years.

| The time spent on social media | Mobility^a^ | Looking after myself | Doing usual activities^a^ | Having pain or discomfort^a^ | Feeling worried, sad or unhappy^a^ | EQ VAS^b^ |
| --- | --- | --- | --- | --- | --- | --- |
| **weekday** |  |  |  |  |  |  |
| < 30 min | 1 (ref) | NA | 1 (ref) | 1 (ref) | 1 (ref) | 1 (ref) |
| 30 min to 2 hours | 0.70 (0.44, 1.10) |  | 1.60 (0.94, 2.71) | 1.25 (0.96, 1.64) | 1.39 (0.99, 1.95) | -1.61 (-3.28, 0.51) |
| 2 to 4 hours | 0.91 (0.56, 1.46) |  | **1.94 (1.13, 3.33)** | **1.51 (1.14, 2.00)** | **1.70 (1.19, 2.41)** | **-2.97 (-4.76, -1.19)** |
| 4 to 6 hours | 0.96 (0.46, 2.00) |  | **2.40 (1.18, 4.90)** | **1.89 (1.26, 2.85)** | **2.05 (1.26, 3.35)** | **-5.12 (-8.10, -2.15)** |
| > 6 hours | **2.21 (1.05, 4.65)** |  | **4.00 (1.84, 8.71)** | **2.29 (1.37, 3.85)** | **2.08 (1.13, 3.85)** | **-7.70 (-11.82, -3.59)** |
| **Weekend day** |  |  |  |  |  |  |
| < 30 min | 1 (ref) | NA | 1 (ref) | 1 (ref) | 1 (ref) | 1 (ref) |
| 30 min to 2 hours | 0.97 (0.52, 1.82) |  | 1.12 (0.61, 2.08) | 1.31 (0.93, 1.85) | 0.89 (0.61, 1.32) | -1.58 (-3.67, 0.50) |
| 2 to 4 hours | 1.24 (0.68, 2.27) |  | 1.39 (0.77, 2.53) | **1.48 (1.05, 2.07)** | 1.15 (0.79, 1.68) | **-2.60 (-4.66, -0.55)** |
| 4 to 6 hours | 0.54 (0.23, 1.29) |  | 1.75 (0.89, 3.47) | **1.78 (1.20, 2.65)** | 1.17 (0.74, 1.86) | **-4.56 (-7.22, -1.90)** |
| > 6 hours | **2.42 (1.17, 5.02)** |  | **2.19 (1.05, 4.54)** | **2.50 (1.60, 3.89)** | **1.91 (1.17, 3.13)** | **-5.90 (-9.09, -2.71)** |

The table is based on an imputed data set.

Bold print indicates statistical significance.

All models were adjusted for child’s age, sex, ethnic background, family composition, maternal educational level, and net household income.

a Values represent odds ratios and 95% CI derived from logistic regression analyses.

b Values represent beta coefficients and 95% CI derived from linear regression analyses.
